# Community Awareness Toward Multiple Sclerosis in the Western Region of Saudi Arabia: A Cross-Sectional Study

**DOI:** 10.7759/cureus.28689

**Published:** 2022-09-02

**Authors:** Maryam Dahlawi, Manar A Ghazzawi, Shahd M Alharthi, Elaf A Yanksar, Muhjah M Almurakshi, Fayrouz R Khatteb, Ruqayya Azher, Motasim Jawi, Rami Algahtani

**Affiliations:** 1 Medicine, Umm Al-Qura University, Makkah, SAU; 2 Community Medicine, Umm Al-Qura University, Makkah, SAU; 3 Physiology, College of Medicine, University of Jeddah, Makkah, SAU; 4 Internal Medicine, College of Medicine, Umm Al-Qura University, Makkah, SAU

**Keywords:** western region, saudi arabia, knowledge, awareness, neurological disease, multiple sclerosis

## Abstract

Objectives: The aim of this study is to assess the awareness level and knowledge about multiple sclerosis (MS) disease among the general population in the Western region of Saudi Arabia.

Methods: This study was a community-based cross-sectional descriptive study carried on by an online questionnaire, previously validated in published studies, to all residents in the western region of Saudi Arabia who successfully fulfilled the inclusion and exclusion criteria a total number of 4038.

Results: Out of the total number of respondents (n=3,536), the majority 46% (1,625) showed a low level of knowledge, while 31% (1,116) have an average level of knowledge, and 22.5% (795) have a high level of knowledge. Various factors including age, gender and socioeconomic status showed a potential association.

Conclusion: This community-based survey showed a low level of knowledge in regard to MS in the Western region of Saudi Arabia. Multiple variables showed potential associations that can be utilized to efficiently direct governmental and non-governmental health organizations' efforts to maximize awareness of this condition to aid early recognition and early treatment in the hope of better outcomes.

## Introduction

Multiple sclerosis (MS) is a lifelong condition considered a main and most prevalent inflammatory neurodegenerative disease affecting the central nervous system (CNS) [1-3]. MS is considered to be an organ-specific disease with immune-mediated myelin destruction associated with the term autoimmunity, mediated by autoreactive T helper (Th)1 and Th17 cells [4-6]. It is affecting more than two million people worldwide and is currently incurable, no medication fully prevents or reverses progressive neurological deterioration [7-10]. MS patients present with variable signs and symptoms. Different episodes including optic neuritis, and spinal cord syndrome were common that may lead to serious complications, progressive disability, and even death [11].

Multiple epidemiological studies showed a high prevalence of MS in Saudi Arabia and the number of cases continues to rise [1,12]. Further studies are needed to evaluate community-specific risk factors related to increasing prevalence in Saudi Arabia. 

Previous studies in Saudi Arabia [13], including several cities such as Riyadh city [14], Qassim region [1], and Taif city [11], have explored the awareness of MS among the general population. As it's a crucial part of early diagnosis and treatment with disease-modifying medications, and in turn improves outcomes. The conclusion of these studies showed a consistent lack of knowledge in these regions. Because of this lack of awareness, we have to expand the studies on the MS level of knowledge within the remaining cities in Saudi Arabia. Given the aforementioned results, we conducted this study to assess the level of awareness and knowledge about MS among the general population in the Western region of Saudi Arabia.

## Materials and methods

Study design

This study was a community-based cross-sectional descriptive study conducted in the region of Makkah, Kingdom of Saudi Arabia at Umm AlQura University (UQU), 2021 to 2022. 

Study population and sampling methodology

The general population resides in the western region of Saudi Arabia. Adults aged 18 years and more who agreed to participate in the study were included. Any healthcare worker, health specialties students, or MS patients who were diagnosed to have MS of all types of all ages and nationalities were excluded. Data were collected through a previously validated online questionnaire in published studies [1,6]. It was formulated in Arabic and English, was completed using Google Documents, and distributed electronically via social media applications. There was a total of 4,038 participants.

The questionnaire covered the following sections: A) The participants' sociodemographic data, including age in years, gender, nationality, residence, education, marital status, total perceived family income per month, occupation and source of information about MS. B) Level of participants' knowledge and awareness about MS. The questionnaire included 15-item, close-ended questions.

Participants were asked to respond to knowledge items as either yes or no. Incorrect responses were given a score of zero, and correct answers were assigned a score of one. Knowledge percentage scores were assigned based on the literature. The knowledge levels had three components, a low level of knowledge for less than 33% of possible correct answers, an average level of knowledge for 33%-65% of possible correct answers, and a high level of knowledge for 66%-100% of possible correct answers.

Data analysis

Data were analyzed by the Statistical Package for Social Sciences (SPSS®) software for Mac, version 26 (IBM Corp., Armonk, NY, USA). For numerical variables analysis, we used the t-test and chi-square test for categorical data analysis. A p<0.05 value was considered statistically significant. Additionally, an adjusted odd ratio with a 95% confidence interval was reported.

Ethical considerations

Ethical approval was provided by the institutional review board (IRB) of UQU (No. HAPO-02-k-012-2021-09-759). Consent was obtained electronically from all participants after the study aims were explained.

## Results

Socio-demographics data

The total responses to the questionnaire were 4,038. Of which, 502 were excluded because they did not meet the inclusion criteria, as 164 were healthcare workers, 302 were health specialties students, and 36 of the participants were MS patients who were diagnosed with the disease, the remaining total sample 3,536 were included in the analysis. 

Table 1 shows that 1,543 (43.6%) were from the age group (18-25) years, 1,073 (30.3%) were (26-39) years, and the remaining 920 (26.0%) were 40 years and above. Most participants were female 2,292 (64.8%), and 3,266 (92.4%) were Saudi in nationality. 879 (24.9%) of respondents were from Makkah city, 848 (24.0%) were from Jeddah city, 589 (16.7%) were from Madina El Monawara, 510 (14.4%) from Al-Qunfudhah, 461 (13.0%) from Taif city, 166 (4.7%) were from Rabigh, the rest of the participants were from Jamum, Laith, and Bahrah. Regarding the education level, more than half of the participants 2,055 (58.1%) had bachelor’s degrees and 823 (23.3%) had high school degrees. 1,759 (49.7%) of the participants about were single, 1,625 (46.0%) were married and the rest were between divorced and widows. Respecting the total family income perceived per month, 1,445 (40.9%) of participants perceive more than 10,000 Saudi riyals per month, 1,296 (36.7%) perceive between 5,000 and 10,000, and the remaining 795 (22.5%) perceive less than 10,000 Saudi riyals per month. 1,175 (33.2%) participants were employed as non-healthcare workers, 1,508 (42.6%) were unemployed, and the rest of the participants 853 (24.1%) were non-health specialties students. The majority of the participants had heard about MS before, about 2306 (65.2%), the source of information was from the internet and social media for 915 (25.9%) of them, 785 (22.2%) had their information from MS patients, 194 (5.5%) received information from family, friends or neighbors, and 162 (4.6%) from health care workers. The remaining sources were TV, radio, newspaper, posters, leaflets, and brochures. 114 (2.8%) of them have combined sources for MS information.

**Table 1:** Demographic characteristics of respondents

Characteristic	Category	Frequency and percentage
Age (Years)	18-25 years	1,543 (43.6%)
	26-39 years	1,073 (30.3%)
	40 years and above	920 (26.0%)
Gender	Female	2,292 (64.8%)
	Male	1,244 (35.2%)
Nationality	Saudi	3,266 (92.4%)
	Non-Saudi	270 (7.6%)
Residence	Makkah	879 (24.9%)
	Jeddah	848 (24.0%)
	Taif	461 (13.0%)
	Madina El Monawara	589 (16.7%)
	Al-Qunfudhah	510 (14.4%)
	Laith	52 (1.5%)
	Bahrah	25 (0.7%)
	Rabigh	166 (4.7%)
	Jamum	6 (0.2%)
Education	High school	823 (23.3%)
	Diploma	305 (8.6%)
	Bachelor’s degree	2,055 (58.1%)
	Postgraduate degree	244 (6.9%)
	Other	109 (3.1%)
Martial status	Married	1,625 (46.0%)
	Single	1,759 (49.7%)
	Widow	54 (1.5%)
	Divorced	98 (2.8%)
Total perceived family income per month	Less than 5000	795 (22.5%)
	5000-10000	1,296 (36.7%)
	More than 10000	1,445 (40.9%)
Occupation	Employed	1,175 (33.2%)
	Unemployed	1,508 (42.6%)
	Student	853 (24.1%)
If heard before about Multiple Sclerosis	Yes	2,306 (65.2%)
	No	1,230 (34.8%)
Source of information	Internet or social media.	915 (25.9%)
	MS patient.	785 (22.2%)
	Family, friends or neighbors.	194 (5.5%)
	Newspaper.	82 (2.3%)
	TV or radio.	79 (2.2%)
	MS information leaflets, brochures, posters etc.	72 (2.0%)
	Health care workers.	162 (4.6%)
	Combined sources.	8 (0.2%)
	Others .	712 (20.1%)
	I didn't hear about the disease	527 (14.9%)

Level of knowledge of respondents

Table 2 describes the responses of the participants toward MS knowledge items, most of the participants 2,658 (75.2%) know that MS is a neurological disease resulting from an immunological disorder. 1,992 (56.3%) of the participants had answered the question correctly and agreed that MS is not a hereditary disease that has genetic causes. When asked about whether pollution could be a cause of MS 2,267 (64.1%), they had answered no, and more than half of the participants 1,808 (51.1%) knew that age is a risk factor for MS. 2,068 (58.5%) of participants agreed that young age is mostly affected by MS. Lower than half of participants, 1,624 (45.9%) agreed that MS affects women more than males. The majority of the participants 2,990 (84.6%) answered the question that asks if you can get MS disease from someone else correctly, 2,845 (80.5%) agreed that MS is a neurological disease with stages of exacerbation and remission. 71.9% chosen yes MS can cause disability complications, while just 970 (27.4%) knew that lifestyle does not decrease disability in MS more than treatment, half of participants 1,723 (51.3%) answered yes there is a relationship between living in hot or cold areas and having MS systems complications. MS is not a curable disease and drugs can keep the disease from getting worse for a while, 2,286 (64.6%) chose the right answer. Only a few participants 1,230 (34.8%) knew that a specific diet is not required for the treatment of MS, while the majority of them 2,678 (75.7%) answered the last question correctly.

**Table 2:** Responses of respondents to MS knowledge items

		Yes (N%)	No (N%)
1	MS is neurological diseases result from immunological disorder	2,658 (75.2%)	878 (24.8%)
2	MS is hereditary disease that have genetic causes	1,544 (43.7%)	1,992 (56.3%)
3	Pollution could be a cause of MS	1,269 (35.9%)	2,267 (64.1%)
4	Age is a risk factor for MS	1,808 (51.1%)	1,728 (48.9%)
5	Young age is mostly affected	2,068 (58.5%)	1,468 (41.5%)
6	MS affect female more than male	1,624 (45.9%)	1,912 (54.1%)
7	Can you get ms disease from someone else	546 (15.4%)	2,990 (84.6%)
8	MS is neurological disease with stages of exacerbation and remission	2,845 (80.5%)	691 (19.5%)
9	MS cause disability complication	2,542 (71.9%)	994 (28.1%)
10	Lifestyle decrease disability in MS more than treatment	2,566 (72.6%)	970 (27.4%)
11	Is there a relationship between living in hot or cold areas and having MS systems complication	1,723 (48.7%)	1,813 (51.3%)
12	MS is not a curable disease. Drug can keep the disease from getting worse for a while	2,286 (64.6%)	1,250 (35.4%)
13	Specific diet is required for the treatment of MS	2,306 (65.2%)	1,230 (34.8%)
14	There is role for vitamin D and ultraviolet ray of sun in protection against MS	2,678 (75.7%)	858 (24.3%

**Figure 1:**
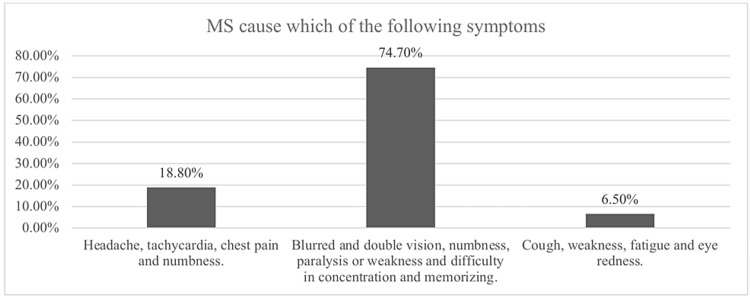
Responses of respondents to multiple sclerosis symptoms knowledge items

Figure 1 shows the majority of respondents about 2,641 (74.7%) had chosen MS symptoms as blurred and double vision, numbness, paralysis or weakness and difficulty in concentration and memorizing which is the correct answer. 666 (18.8%) their choice of symptoms were headache, tachycardia, chest pain and numbness. The rest of the participants who were 229 (6.5%) had chosen cough, weakness, fatigue and eye redness.

**Figure 2:**
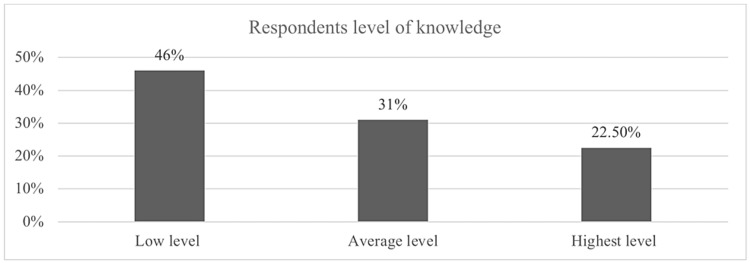
Level of knowledge of respondents

Figure 2 shows that the majority of respondents who were 1,625 (46%) have a low level of knowledge, while 1,116 (31%) have an average level of knowledge, and 795 (22.5%) have a high level of knowledge.

The relationship between the level of participants' knowledge and awareness regarding MS and demographic characteristics

Table 3 describes the relationship between the demographic characteristics of respondents and their level of knowledge of MS. For the relationship between the age of participants and level of knowledge, the age group is from 18-25 years, 696 of them had a low level of knowledge, 505 had an average level of knowledge, and 345 of them had a high level of knowledge. As for the group from 26 to 39 years, 527 had a low level of knowledge, 312 had an average level of knowledge, and 234 of them had a high level of knowledge. The majority of female participants about 1,058 had a low level of knowledge as well as most of the participants of Saudi nationality (1,519) had a low level of knowledge, and 728 of them had a high level of knowledge. 403 of the participants who lived in Makkah had a low level of knowledge, 272 had an average level of knowledge, and 204 had a high level of knowledge. 882 participants who lived in Jeddah, Taif, and Medina ElMonawara had low levels of knowledge, 604 of them had an average level of knowledge, and 412 of them had a high level of knowledge. 978 bachelor's degree participants had a low level of knowledge, 643 had an average level of knowledge, and 434 of them had a high level of knowledge. 768 married participants and 803 single ones had a low level of knowledge while 360 married and 389 single participants had a high level of knowledge. The majority of participants had more than 10,000 as a total family income per month, and 676 of them were considered to have a low level of knowledge. Regarding occupation, the majority of participants are unemployed, and 682 of them had a low level of knowledge. There is a significant relationship between the level of awareness and whether they had previously heard about MS (p=0.003), 2,306 participants had heard about MS previously, and 501 of them were considered to have a high level of knowledge. Internet and social media are considered to be the major source of information for MS, 202 out of 915 of those who had chosen this as a source of information had a high level of awareness. While 785 participants had chosen MS patients as a source of information about the disease, 168 of them had a high level of knowledge.

**Table 3:** Relationship between demographic characteristics and level of knowledge toward multiple sclerosis

Characteristic	Category	Low level of knowledge	Average level of knowledge	Highest level of knowledge	P-value
Age (Years)	18-25 years	696	502	345	0.226
	26-39 years	527	312	234	
	40 years and above	414	295	211	
Gender	Female	1058	710	524	0.583
	Male	579	399	266	
Nationality	Saudi	1519	1019	728	0.556
	Non-Saudi	118	90	62	
Residence	Makkah	403	272	204	0.759
	Jeddah	395	269	184	
	Taif	199	161	101	
	Madina El Monawara	288	174	127	
	Al-Qunfudhah	244	158	108	
	Laith	25	13	14	
	Bahrah	14	8	3	
	Rabigh	66	52	48	
	Jamum	3	2	1	
Education	High school	372	257	194	0.272
	Diploma	123	105	77	
	Bachelor’s degree	978	643	434	
	Postgraduate degree	116	68	60	
	Other	48	36	25	
Martial status	Married	768	497	360	0.731
	Single	803	567	389	
	Widow	23	17	14	
	Divorced	43	28	27	
Total perceived family income per month	Less than5000	380	240	175	0.807
	5000-10000	581	421	294	
	More than10000	676	448	321	
Occupation	Employed	543	374	258	0.865
	Unemployed	682	480	346	
	Student	412	255	186	
If heard before about Multiple Sclerosis	Yes	1115	690	501	0.003
	No	522	419	289	
Source of information	Internet or social media.	448	265	202	0.057
	MS patient.	382	235	168	
	Family, friends or neighbors.	89	55	50	
	Newspaper.	36	24	22	
	TV or radio.	41	25	13	
	MS information leaflets, brochures, posters etc.	36	18	18	
	Health care workers.	81	45	36	
	Combined sources.	3	3	2	
	Others .	281	257	174	
	I didn't hear about the disease	240	182	105	

## Discussion

Recent epidemiological studies showed increasing rates of MS in Saudi Arabia and the number of cases continues to rise, reflecting the advances in diagnostic modalities and easier access to specialized centers [1]. Currently, all forms of MS have disease-modifying therapeutics that alter the course of their disease. Although treatable, MS has a catastrophic personal, social, economic, and psychological sequel. Early diagnosis and implementation of these agents are essential to improve outcomes and minimize disability [15,16]. Community awareness is paramount in such conditions to seek specialized physicians and allow early diagnosis.

Previous epidemiological studies showed a low level of knowledge regarding MS in multiple regions of Saudi Arabia [13]. The current research was conducted to assess the level of awareness of MS among the population of the Western region of Saudi Arabia. The survey was inclusive of all adults above the age of 18, and 43.6% of respondents were from the age group (of 18-25) years, which may reflect easier access to online resources. Most respondents were females (65%), which goes in line with other regional studies within the kingdom [1,11].

Higher percentages of the participants recognized that MS affects the nervous system (75.2%), has different stages (80.5%), has a relation to vitamin D (75.7%), is treatable (64.6%), and disabling ( 71.9%). MS can present in variable presentations, specific symptoms like visual symptoms, paresthesia, and weakness are the most common. 75% of the study, participant were able to identify these symptoms as MS symptoms which is helpful as the relatively mild symptoms and the remitting nature of MS attacks may delay seeking medical care.

We found a significant relationship between the level of awareness and whether they had previously heard about MS with a significant p-value (p=0.003), 65.2% participants had heard about MS previously, and 21.73% considered to have a high level of knowledge. These findings are consistent with the findings of the study among the Saudi population in Taif city [11] and are likely reflecting scientific curiosity toward an uncommon disease with potential fearsome outcomes. Most of the knowledge learnt about MS was obtained from online sources making a great opportunity to spread more organized, regulated, and factual awareness materials. In addition, Arhan et al. [17] found written materials to be an effective and easy-to-implement methods to improve the understanding of a condition for the patient and their family. Multiple approaches including online sources, written materials and community events will certainly improve the understanding of the disease and the overall awareness of the community.

The fact that more than half of the participants had a degree in higher education (59%) did not improve the level of awareness of the community. In addition, we found no significant association between the level of knowledge and sociodemographic data, including age, gender, nationality, level of education, income, or occupation. This was consistent with previous studies, except Al-Batanony et al., where female participants had a higher level of knowledge.

We suggest arranging health education programs and campaigns regarding MS, they are essential for enhancing the community awareness level in order for the early detection and proper management of this serious disease in the hope of better outcomes. As our result showed that most of the knowledge learnt about MS was obtained from online sources therefore utilizing social media and other online channels is important to improve awareness. Further research regarding community awareness of MS is needed to dissect the causes of mis-knowledge on a larger scale inclusive of the entire kingdom to assist the current efforts and maximize its benefits.

Study limitations

We are aware of a few study limitations that should be addressed. The study collected data using an online questionnaire, which may affect its validity if the answers were researched. The population of the Western region of Saudi Arabia is not high enough to represent the population of Saudi Arabia, hence the results cannot be generalized to the rest of the kingdom.

## Conclusions

Saudi Arabia is a large country and community knowledge of MS is variable across different areas. Previous reports showed a low level of knowledge in various geographic areas. This community-based survey showed a low level of knowledge in the Western region of Saudi Arabia. Multiple variables showed potential associations that can be utilized to efficiently direct governmental and non-governmental health organizations' efforts to maximize awareness of this condition to aid early recognition and early treatment in the hope of better outcomes as multiple therapeutic diseases modifying options are readily available.
